# Multinational Association of Supportive Care in Cancer (MASCC) expert opinion/guidance on the use of clinically assisted hydration in patients with advanced cancer

**DOI:** 10.1007/s00520-024-08421-6

**Published:** 2024-03-13

**Authors:** Jo Hayes, Eduardo Bruera, Gregory Crawford, Mapi Fleury, Marcos Santos, Jo Thompson, Andrew Davies

**Affiliations:** 1 St. Catherine’s Hospice, Crawley, UK; 2 The University of Texas MD Anderson Cancer Center, Houston, USA; 3 University of Adelaide and Northern Adelaide Local Health Network, Adelaide, Australia; 4 Department of Oncology, CHUV, Lausanne University Hospital, Lausanne, Switzerland; 5 CONFIAR Radiotherapy, Goiania, Brazil; 6 Royal Surrey County Hospital, Guildford, UK; 7 Trinity College Dublin, University College Dublin, Our Lady’s Hospice, Dublin, Ireland

**Keywords:** Clinically assisted hydration, Neoplasms, Palliative care, Practice guideline, Advanced cancer

## Abstract

**Purpose:**

The provision of clinically assisted hydration (CAH) in patients with advanced cancer is controversial, and there is a paucity of specific guidance and so a diversity in clinical practice. Consequently, the Palliative Care Study Group of the Multinational Association of Supportive Care in Cancer (MASCC) formed a sub-group to develop evidence-based guidance on the use of CAH in patients with advanced cancer.

**Methods:**

This guidance was developed in accordance with the MASCC Guidelines Policy. A search strategy for Medline was developed, and the Cochrane Database of Systematic Reviews and the Cochrane Central Register of Controlled Trials were explored for relevant reviews/trials, respectively.

**Results:**

Due to the paucity of evidence, the sub-group was not able to develop a prescribed guideline, but was able to generate a number of “expert opinion statements”: these statements relate to assessment of patients, indications for CAH, contraindications for CAH, procedures for initiating CAH, and reassessment of patients.

**Conclusions:**

This guidance provides a framework for the use of CAH in advanced cancer, although every patient requires individualised management.

## Introduction 

It is not uncommon for patients with advanced cancer to develop problems that impact on their ability to ingest and/or absorb enough fluids to maintain hydration: such problems may be acute and necessitate short-term fluid therapy (e.g. vomiting, diarrhoea) or chronic and necessitate ongoing fluid therapy (e.g. dysphagia, malignant bowel obstruction). Although such problems can occur at any stage, they are especially common in patients in the terminal phase (i.e. last days to weeks of life) [1].

In general, clinically assisted hydration (CAH) is used to manage dehydration/maintain hydration, and in many patients with advanced cancer, the decision about initiating CAH is not difficult/contentious. However, the provision of CAH in the terminal phase is one of the most contentious issues in medicine [2, 3]. The reasons for contention include the following: (a) the lack of evidence for/against CAH [4, 5]; (b) the disparate opinions of healthcare professionals about CAH [6]; and (c) the generally positive opinions of patients and their carers about CAH (and the generally negative opinions about withholding/withdrawing CAH), in this specific clinical scenario [6, 7].

Unsurprisingly, the provision of CAH in the terminal phase is extremely variable within clinical practice (12–88% of cancer patients in the last week of life) [8]. In some cases, the decision appears relatively straightforward, with the patient having a clear indication for CAH (e.g. malignant hypercalcaemia) or a clear contraindication to CAH (e.g. decompensated cardiac failure). However, in many cases, the decision is much more subjective. Hence, the Palliative Care Study Group of the Multinational Association of Supportive Care in Cancer (MASCC) formed a sub-group to develop evidence-based guidance on the use of CAH in patients with advanced cancer.

At the time the sub-group started the project, there were no up-to-date international guidelines on the clinical aspects of CAH in patients with advanced cancer, although there are older guidelines relating to this cohort of patients [9] and some relevant national guidelines, e.g. Japanese Society for Palliative Medicine guidelines [10]. In contrast, there are multiple publications relating to the ethical issues surrounding CAH (reflecting the limited evidence to guide clinical practice and the clinical relevance of the subject) [11, 12].

## Background

### Definitions

For the purposes of this guidance, CAH “refers to the practice of providing fluids in the form of a drip, usually either intravenously or subcutaneously (a process known as hypodermoclysis) or via a nasogastric tube or gastrostomy to prevent dehydration. It does not include assisting a person to drink via the oral route” [13]. Synonymous terms within the medical literature include “medically assisted hydration” [4] and “artificial hydration” [11]. As already stated, hypodermoclysis refers to fluids given via the subcutaneous route, whilst proctoclysis refers to fluids given via the rectal route (which is another alternative) [14]. Importantly, CAH is utilised in the management of dehydration as well as in the prevention of dehydration.

Other definitions used in this guidance include “advanced cancer” (i.e. “cancer that is unlikely to be cured or controlled with treatment”) [15], “end-of-life” (i.e. the last year of life) [16], and “terminal phase” (i.e. the last days to weeks of life) [17]. Importantly, patients with advanced cancer may not be at the end-of-life (as defined), and prognostication remains exceptionally challenging (especially when the prognosis is of the order of months to years rather than days to weeks) [18]. Also, the trajectory of the illness may change (speed up or slow down), and/or acute events may intervene (cancer-related or otherwise).

### Fluid requirements

Water makes up ~ 60% of human body weight, which equates to 42 l in a 70-kg man [19]. Two-thirds (28 l) of the water is intracellular, and one-third (14 l) of the water is extracellular: the extracellular water includes 10 l of interstitial fluid (including lymph), 3 l of plasma, and 1 l of “transcellular” fluid (i.e. cerebrospinal fluid, ocular fluid, pleural fluid, peritoneal fluid, synovial fluid). Normally, homeostatic mechanisms maintain relatively constant levels of body water (± 0.2% body weight during a 24-h period) [19]. Body water (specifically intracellular water) decreases with aging: water makes up ~ 50% of human body weight in an 80-year-old man [20]. Table 1 shows the typical sources of water intake and water loss in sedentary adults in temperate climates [19].

**Table 1:** Water intake/loss in sedentary adults in a temperate climate [19]

**Sources of water intake**	**Volume**	**Comment**
Drinks	1575 ml/day (range, 1400–1750 ml/day)	70–80% total intake
Food	675 ml/day (range, 600–750 ml/day)	20–30% total intake
Metabolism	300 ml/day (range, 250–350 ml/day)	
Total	2550 ml/day (range, 2250–2850 ml/day)	Lower end of range relates to sedentary females; upper end of range relates to sedentary males
**Sources of water loss**	**Volume**	**Comment**
Urine	1600 ml/day (range: 1200–2000 ml/day)	
Skin (“insensitive perspiration”)*	450 ml/day	Dependent of climate, air temperature, and relative humidity
Respiration	300 ml/day (range, 250–350 ml/day)	Dependent of climate, air temperature, and relative humidity
Faeces	200 ml/day (range, 100–300 ml/day)	
Total	2550 ml/day (range, 2000–3100 ml/day)	

*Pyrexia and exercise result in activation of sweat glands/additional water loss (“sensitive perspiration”)

### Fluid homeostasis

As discussed, homeostatic mechanisms normally maintain relatively constant levels of body water (± 0.2% body weight during a 24-h period) [19]. The following discussion is a simplified explanation of the main homeostatic mechanisms. If water loss exceeds water intake, then there is an increase in ECF osmotic pressure (plasma osmolality), which is sensed by osmoreceptors in the hypothalamus, which results in increased secretion of antidiuretic hormone (ADH) from the pituitary, which in turn results in increased reabsorption of water by the kidneys (and in the sensation of thirst). It should be noted that urinary concentrating ability decreases with aging, and this is one of the factors linked to an increased incidence of dehydration in the elderly [20]. The increase in osmotic pressure separately results in the sensation of thirst. However, thirst sensation may be blunted in some older persons (which is another factor linked to an increased incidence of dehydration in the elderly) [21]. Similarly, thirst sensation may be blunted in some (but not all) patients with advanced cancer [22, 23].

### Dehydration

Water is vital for life, and inevitably abstinence results in death from dehydration within a few days in temperate climates. There are three types of dehydration: (1) isotonic dehydration - equal loss of salt and water (e.g. profuse diarrhoea); (2) hypertonic dehydration - water loss is greater than salt loss (e.g. reduced intake); and (3) hypotonic dehydration - salt loss is greater than water loss (e.g. replacement of “lost” fluid with water or other fluids that have relatively less sodium and/or potassium) [19].

Currently, there are no universally accepted diagnostic criteria for dehydration [24]. Table 2 shows the “reliability and accuracy” of different clinical and laboratory indicators of dehydration, and none of them reaches the threshold for “high” reliability and accuracy. Other (research rather than clinical) methods for assessing hydration/dehydration status include bioelectrical impedance analysis (and related bioelectrical impedance vector analysis [25]), isotope dilution techniques, and neutron activation analysis [24].

**Table 2:** Indicators of dehydration [24]

Indicator	“Reliability and accuracy”
**Symptoms**	
Thirst	Medium
**Signs**	
Postural hypotension, ≥ 20 mmHg	Medium to high
Reduced systolic BP (seated), ≤ 100 mmHg	Medium to high
Dark urine	Medium
Dry mucous membranes	Low
Dry eyes (absence of tears)	Low
Dry axilla	Low
Sunken eyes	Low
Reduced skin turgor	Low
**Laboratory investigations**	
Blood urea nitrogen/creatinine ratio, ≥ 20	Medium to high
Blood osmolality, > 300 mmol/kg	Medium to high
Serum sodium, high	Medium
Mean corpuscular volume, high	Medium
Haematocrit/haemoglobin ratio, high	Medium
Urine specific gravity, ≥ 1.025	Medium to high
Urine osmolality, ≥ 800 mmol/kg	Medium to high

Importantly, many patients with advanced cancer experience the symptom of xerostomia (“the subjective sensation of dryness of the mouth”) [26], which is very different from the symptom of thirst (“the uneasy or painful sensation caused by want of drink”) [27], but which is often misconstrued as being analogous problems. Thus, although most patients with dehydration will report xerostomia, only a very small proportion of patients with xerostomia will be clinically dehydrated. The latter is important both in terms of assessment of patients, but also in terms of appropriate treatment of these symptoms [28].

### Clinically assisted hydration

As discussed above, there is a lack of evidence for/against CAH [4, 5]. The purported positive effects of CAH include provision of a basic human requirement, maintenance of patient comfort (e.g. prevention of thirst, prevention of dry mouth), maintenance of renal perfusion/function (e.g. prevention of delirium, prevention of opioid toxicity), and prolongation of life [29]. In contrast, the purported negative effects of CAH include “medicalisation” of death, problems due to fluid overload (e.g. worsening of peripheral oedema, worsening of ascites), problems due to fluid-related conditions (e.g. worsening of vomiting, worsening of respiratory secretions), and prolongation of the dying process [29]. In addition, it has been claimed that ketones and other by-products of dehydration can have positive effects on the patients’ condition/symptom control (i.e. analgesic effects, sedative effects).

Higashiguchi et al. [10] reviewed the evidence for CAH causing/exacerbating certain fluid overload problems (i.e. peripheral oedema, ascites, pleural effusion). It should be noted that the data came from observational studies rather than interventional studies (since the latter had not assessed these adverse events). Higashiguchi et al. reported that such fluid overload problems may be exacerbated by higher volumes of fluid (i.e. > 1000 ml/day), and this was reflected in their related guidelines for parenteral fluid management in “terminal cancer patients” [10].

The recently updated Cochrane systematic review of medically assisted hydration (MAH) for adult palliative care patients [4] identified four randomised controlled trials/RCTs [30–33]. The authors of this systematic review concluded that “in adults receiving palliative care in the end stage of their illness, there remains insufficient evidence to determine whether MAH improves QoL *(quality of life)* or prolongs survival, compared with placebo or standard care” [4].

The initial three RCTs involved patients with dehydration, and the patients were treated with a fixed, relatively low, volume of fluid (i.e. 1 l/day) [30–32]. However, clinical signs of “mild” dehydration occur with a loss of fluid equivalent to 3–5% of body weight (equivalent to 2–3.5 l for a 70-kg person) [34], and so many participants are likely to have remained dehydrated during the study period. The subsequent RCT excluded patients with dehydration, and the patients were treated with a variable (weight-dependent) volume of fluid [33]: the volume of fluid was derived from generic fluid therapy guidance from the National Institute for Health and Care Excellence (NICE) (UK) [35]. It should be noted that the latter was a feasibility study (which achieved all of its criteria for success) and that the related definitive study is currently recruiting participants [36].

Recently, Pérez-Camargo et al. reported a RCT comparing low-volume CAH (500 ml/day) with or without supplemental vitamins and trace elements in clinically dehydrated patients with various symptoms (i.e. pain, fatigue, anorexia, chronic nausea, somnolence, insomnia, dyspnoea, “lack of overall wellbeing”, anxiety, depression) [37]. The trial lasted for 4 weeks, and patients in the CAH alone arm reported no improvement in symptoms, whilst patients in the CAH with vitamins and trace elements reported improvement in a number of symptoms (and a statistically significant improvement in pain and anorexia).

CAH is considered a medical treatment, and recent European Society for Clinical Nutrition and Metabolism (ESPEN) guidelines highlight the ethical principles regarding the provision/omission of CAH (Box 1) [11]. The ESPEN guideline is based on universal ethical principles (i.e. autonomy, beneficence, non-maleficence, justice), but readers are encouraged to check their own national guidance on the provision/omission of CAH and analogous treatments.

## Methods

The aim of the sub-group was to develop comprehensive, clinically relevant, evidence-based guidance on the provision of CAH in patients with advanced cancer. Thus, it was agreed that the proposals could include ones supported by “high” levels of evidence (e.g. systematic reviews), as well as ones supported by “low” levels of evidence (e.g. expert opinion), if the topic was deemed to be clinically relevant.

This guidance was developed in accordance with the MASCC Guidelines Policy [38]. The sub-group adopted the National Cancer Institute (NCI) definition of advanced cancer (see above) [15], and data was included from studies involving cancer patients still receiving anti-cancer treatment, as well as cancer patients receiving palliative care alone.

A search strategy for Medline was developed (Appendix), and the Cochrane Database of Systematic Reviews and the Cochrane Central Register of Controlled Trials (CENTRAL) were explored for relevant reviews/trials, respectively. The review of the published literature was restricted to papers written in English and to papers relating to adult (≥ 19 years) humans.

**Box 1:** Ethical considerations relating to provision of CAH in patients with advanced cancer [11]

• The physician/multidisciplinary team has the ultimate responsibility for making the decision on CAH.
• CAH should be considered if the potential benefits outweigh the potential burdens (and vice versa).
• CAH should be considered if it is unclear whether the potential benefits outweigh the potential burdens (i.e. give a trial of CAH).
• The patient does not have the right to demand CAH.
• The patient does have the right to refuse CAH (if the patient has capacity/competence).
• A valid advance directive to refuse treatment must be followed (if the patient does not have capacity/competence).
• The family does not have the right to demand CAH.

All abstracts identified by the search of Medline (1946 to 23rd July 2023) were downloaded into a reference management software package. These abstracts were independently assessed for relevance by the two primary authors (AD, JH), and if one author deemed the abstract relevant, then the full text of the article was obtained. These articles were independently assessed for inclusion by the two primary authors. All of the authors were involved in assessing the randomised controlled trials in the CENTRAL, and the two primary authors were involved in assessing the systematic reviews in the Cochrane Database of Systematic Reviews.

Due to the paucity of evidence, the sub-group was not able to develop a prescribed guideline [38], but was able to generate a number of “expert opinion statements”. These statements were initially developed by the two primary authors and subsequently reviewed/amended by all the authors.

## Results

The searches were last undertaken on 23rd July 2023. The Medline search identified 9091 references, and 142 full-text articles were retrieved and reviewed. The search of the Cochrane Central Register of Controlled Trials identified 1352 references, with 12 deemed relevant (one not identified in Medline search). Similarly, the search of the Cochrane Database of Systematic Reviews identified 45 references, with one deemed relevant (not identified in the Medline search). Ten additional articles were identified from the reference lists of the retrieved articles/Cochrane systematic review.

The sub-group proposes 12 expert opinion statements (Table 3).

**Table 3:** Expert opinion statements regarding clinically assisted hydration in patients with advanced cancer

1 - All patients with advanced cancer should be regularly assessed regarding hydration/dehydration
2 - Patients should be practically supported to maintain oral intake
3 - Reversible causes of decreased fluid intake, or increased fluid loss, should be treated
4 - Decisions relating to clinically assisted hydration should be made by an appropriately constituted multidisciplinary healthcare team together with the patient and their family
5 - Clinically assisted hydration should be considered in patients at risk of dying from dehydration before dying from their cancer
6 - Protocols/processes should exist to deal with conflicts over the initiation (or withdrawal) of clinically assisted hydration
7 - Patients receiving clinically assisted hydration should have a hydration care plan which defines the agreed objectives of treatment and the agreed conditions for withdrawal of treatment
8 - Patients should be given fluids via the most appropriate route (for that patient)
9 - Patients who are dehydrated should be given sufficient fluids to reverse the dehydration
10 - Patients who are not dehydrated should be given sufficient fluids to maintain hydration/prevent dehydration
11 - Clinically assisted hydration should be available in all settings, including the home setting
12 - All patients receiving clinically assisted hydration should be regularly reassessed

### Expert opinion statements

1. All patients with advanced cancer should be regularly assessed regarding hydration/dehydration.

All patients with advanced cancer should be regularly assessed to ensure that they are hydrated (and not dehydrated). Assessment involves taking a history (to determine presence/absence of thirst, fluid/food intake, and fluid losses), performing an examination (to look for signs of dehydration, see Table 2), and undertaking appropriate investigations (to look for indicators of dehydration, see Table 2) [24]. In patients with reduced fluid intake or increased fluid losses, it is important to determine the underlying cause(s) and whether there is any potential for reversibility (see statement 3).

Although clearly related, patients require separate assessments for the need for CAH and the need for clinically assisted nutrition [16]. The MASCC Palliative Care Study Group has published an analogous guidance on the use of clinically assisted nutrition in patients with advanced cancer [39]. Anorexia/reduced food intake may be associated with dehydration (due to reduced fluid intake).

2. Patients should be practically supported to maintain oral intake.

Many patients with advanced cancer, especially patients in the terminal phase, require support to maintain their fluid/food intake due to general frailty and/or specific problems (e.g. dysphagia, low mood). Support ranges from encouraging drinking, making drinks, assisting drinking (e.g. repositioning patient, holding cup), use of “drinking aids” (e.g. drinking straws, beakers), and input from a speech and language therapist (where appropriate). It should be noted that there is “no convincing evidence” to support the use of fluid “thickeners” in patients with dysphagia [40].

3. Reversible causes of decreased fluid intake, or increased fluid loss, should be treated.

Patients with advanced cancer may develop a number of problems, which result in reduced fluid intake (e.g. oral pain, dysphagia), increased fluid loss (e.g. diarrhoea), or both (e.g. vomiting): these “hydration impact symptoms/problems” may be related to the cancer, the cancer treatment, or co-morbid conditions. Many of these problems are potentially reversible, and appropriate treatment may or may not negate the need for ongoing CAH (although some patients will require CAH in the short term). It is also important to review the patient’s medication and consider reducing/stopping drugs which may be adding to fluid losses (e.g. diuretics, laxatives).

4. Decisions relating to clinically assisted hydration should be made by an appropriately constituted multidisciplinary healthcare team together with the patient and their family.

The decision whether or not to initiate CAH, and how to provide CAH, depends on a number of factors (Box 2), and so requires input from the oncology team, the supportive care/palliative care team, other specialist teams/services (e.g. gastroenterology, interventional radiology), and the patient and their family.

**Fig. 1:**
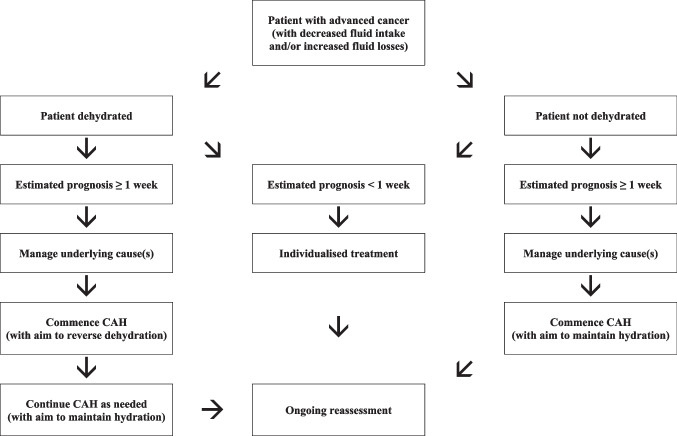
Decision algorithm for CAH in patients with advanced cancer

In patients with a prognosis of weeks to months, the decision to initiate CAH is usually not difficult or controversial, and the main issue relates to the route of administration. However, in patients with a prognosis of days, the decision is often much less straightforward (for the already outlined reasons). Importantly, such decisions must be individualised, and a “blanket” policy of everyone receiving CAH, or nobody receiving CAH, is neither clinically, ethically, nor socially justifiable (Fig. 1).

**Box 2:** Clinical considerations relating to provision of CAH in patients with advanced cancer

• Patient’s views
• (Family’s views)
• Estimated prognosis
• Current hydration status
• Oral intake
• Fluid losses
• Co-morbid conditions (e.g. cardiac disease, renal impairment)
• Suitability of routes of administration
• Availability of indwelling catheters/enteral feeding tubes
• Current/future place of care

5. Clinically assisted hydration should be considered in patients at risk of dying from dehydration before dying from their cancer

The primary indication for CAH in this cohort of patients is the prevention of premature death from dehydration (as opposed to inevitable death from the cancer) (11: Druml et al., 2016). As discussed, the evidence suggests that healthy individuals with no fluid/food intake will die from dehydration within a few days in temperate climates. Thus, our suggestion is that cancer patients with insufficient fluid intake, and an estimated prognosis of ≥ 1 week, should always be considered for CAH. Moreover, our suggestion is that in cases of uncertainty, a trial of CAH should be considered, with precise criteria for continuation/discontinuation (see statement 7) [11].

In patients with an expected prognosis of < 1 week, indications for CAH include “symptom control” (e.g. relief of thirst, management of opioid toxicity) and amelioration of patient and family carer distress relating to withholding/withdrawing CAH. In the latter scenario, there must obviously be no contraindications to the initiation/continuation of CAH.

6. Protocols/processes should exist to deal with conflicts over the initiation (or withdrawal) of clinically assisted hydration.

The provision of CAH is often an emotive subject for patients and their families (particularly in the terminal phase) [6, 7]. As discussed, CAH is a medical treatment, and patients (and/or their families) do not have the right to demand the treatment. In cases of conflict, it is recommended obtaining a second opinion from a suitably qualified healthcare professional: other options such as involvement of a clinical ethics committee or involvement of the legal system are not generally required in this cohort of patients [11].

7. Patients receiving clinically assisted hydration should have a “hydration” care plan which defines the agreed objectives of treatment and the agreed conditions for withdrawal of treatment.

Patients receiving CAH should have a “hydration” care plan which includes the rationale for treatment, the specifics of treatment (e.g. method of CAH), details about ongoing follow-up, details about ongoing reassessment, the indications for continuation of treatment, the indications for discontinuation of treatment, and contact details for relevant healthcare professionals [11].

8. Patients should be given fluids via the most appropriate route (for that patient).

As discussed, CAH can be administered via the enteral, intravenous (IV), or subcutaneous (SC: hypodermoclysis) routes [14]. Enteral administration may be via feeding tubes within the stomach and small intestine or catheters within the rectum (PR: proctoclysis). The choice of route depends on a number of factors, including availability/suitability of the various routes, access to specialist teams/services (e.g. gastroenterology, interventional radiology), current/future place of care, access to relevant supports (e.g. community nurses, family carers), and particularly patient preference.

The IV route is the usual route for administering CAH in the hospital setting, and should always be considered in patients with indwelling intravenous catheters. In other patients, the intravenous route may be indicated (as opposed to the SC route) when large volumes of fluid are required, electrolyte disturbances need correcting, there are skin integrity problems, or there are coagulation problems [41].

The SC route is a well-established route for administering CAH in a variety of settings (e.g. home, hospice) [42–44]. Hypodermoclysis has been repeatedly reported to be effective and generally well tolerated [41]. The main advantages (versus IV route) include ease of usage (requiring minimal training), lower incidence of serious adverse effects (e.g. infection, fluid overload), and lower direct and indirect costs [41].

Various regimens have been used to administer subcutaneous fluids [41, 45]. Non-metal cannulas (22–24 G) are generally recommended and should be changed every 24–48 h to prevent infection/inflammation (or sooner if required): cannulas should be inserted in areas with adequate subcutaneous tissue, and usual sites include the lower lateral abdomen, the upper lateral chest, the scapula, and the upper legs (and to a lesser extent the upper arms). Subcutaneous fluids can be given intermittently or continuously (depending on the volume required): they are generally administered using gravity (rather than with infusion devices), since this technique is simple, effective (with adequate precision), and well tolerated. Different fluids have been used (e.g. 0.9% sodium chloride, 0.18% sodium chloride/4% glucose, 5% glucose), and the choice of fluid depends on the specific clinical situation. Giving sets should be changed every 24 h to prevent infection. Asymptomatic swelling is often seen at the site of the cannula due to the volume being infused, and this is not in itself a reason to re-site the cannula or terminate the infusion.

Early reports of the use of hypodermoclysis within palliative care settings included the use of hyaluronidase to facilitate distribution of the fluid within the connective tissues (and so absorption of fluid into the systemic circulation) [43, 44]. A review of the literature concluded that hyaluronidase is not indicated in most cases [41]. Furthermore, hyaluronidase is relatively expensive (and often difficult to acquire) and has been linked to additional adverse effects (e.g. allergic reactions, fluid overload).

The PR route is an option for administering CAH when other routes are not available/suitable. Proctoclysis has been reported to be effective and generally well tolerated (although there is much less data on this route of administration) [46, 47].

9. Patients who are dehydrated should be given sufficient fluids to reverse the dehydration.

If a patient is dehydrated, and the decision is made to commence clinically assisted hydration, then the goal of treatment should be to reverse dehydration in a timely manner: the volume of fluid, type of fluid (including electrolytes), and rate of infusion should be individualised. Important factors to consider are the degree of dehydration, ongoing fluid losses (i.e. homeostatic, pathological), and relevant co-morbidities (e.g. diabetes mellitus, cardiac problems, renal disease). Routine biochemical testing should be used to guide fluid and electrolyte replacement (except in patients in the terminal phase). Once the patient is rehydrated, an assessment must be made regarding the necessity for ongoing/ “maintenance” treatment (see statement 10).

10. Patients who are not dehydrated should be given sufficient fluids to maintain hydration/prevent dehydration.

If a patient is not dehydrated, and the decision is made to commence clinically assisted hydration, then the goal of treatment should be to maintain hydration: the principles of management in hydrated patients are similar to those in dehydrated patients (although smaller volumes of fluid are generally necessary).

In general, the volume of fluid provided needs to fully match the ongoing fluid losses (i.e. homeostatic, pathological) in order to prevent the occurrence of dehydration. NICE has developed guidance on CAH for adult hospital patients [35]: they recommend 25–30 ml/kg/day of water (with electrolytes/glucose) for maintenance, with lesser volumes (20–25 ml/kg/day) for older persons, and those with frailty, cardiac failure, renal impairment, and malnutrition. The latter guidance is appropriate for many patients with advanced cancer, who have a prognosis of weeks to months, and where the goal of CAH is to maintain hydration (and prevent death due to dehydration).

In the terminal phase, it may be appropriate to use smaller volumes of fluid for “symptom control”, e.g. relief of thirst and management of opioid toxicity: in such cases, the volume of fluid should be titrated to achieve the desired outcome, and this requires an individualised approach.

11. Clinically assisted hydration should be available in all settings, including the home setting.

The provision of CAH for patients with advanced cancer is feasible (and safe) in the home/similar settings, and so a planned discharge from hospital should not be a major factor in the decision to withhold/withdraw relevant treatments. Furthermore, it has the potential for significant cost savings (versus ongoing care in hospital/hospice settings) [48].

CAH has been given subcutaneously (hypodermoclysis) [42, 49–51], intravenously [52], via nasogastric tube/gastrostomy, and rectally (proctoclysis) [46, 47], in the home and similar settings. Importantly, non-professional carers (family) have been able to administer the CAH in these settings (when appropriate and with relevant training) [46, 47, 50, 51].

12. All patients receiving clinically assisted hydration should be regularly reassessed.

All patients receiving CAH should be regularly reassessed with regard to the continuation, amendment, or discontinuation of the relevant treatment [11]. The objectives of reassessment are to (a) ensure the CAH is meeting the patient’s hydration requirements (i.e. that the patient is not under- or over-hydrated), (b) ensure the CAH is well tolerated, (c) ensure the CAH remains acceptable to the patient, and (d) ensure the CAH remains appropriate/consistent with the “goals of care”.

Patients receiving long-term CAH, and especially patients with ongoing GI fluid losses (i.e. vomiting, diarrhoea, high output stomas, small bowel fistulas), require regular biochemical monitoring to guide fluid and electrolyte replacement. Patients in the terminal phase do not require regular biochemical monitoring.

## Conclusion

There is a paucity of evidence for CAH in patients with advanced cancer and so a diversity in clinical practice (especially between hospitals, hospices, and community settings). This guidance provides a framework for the use of CAH in this cohort of patients, although every patient requires thorough assessment and individualised management.

## Data Availability

No datasets were generated or analysed during the current study.

## Appendix EDLINE search strategy

1. Clinically assisted hydration (keyword).

***or 2–17***

2. Medically assisted hydration (keyword).

3. Fluid therapy (MESH term).

4. Fluid management (keyword).

5. Hydration (keyword).

6. Dehydration (MESH term).

7. Rehydration (keyword).

8. Infusions parenteral (MESH term).

9. Intravenous fluids (keyword).

10. Infusions intravenous (MESH term).

11. Subcutaneous fluids (keyword).

12. Infusions subcutaneous (MESH term).

13. Hypodermoclysis (MESH term).

14. Rectal fluids (keyword).

15. Rectal infusions (keyword).

16. Infusions rectal (keyword).

17. Proctoclysis (keyword).

**and**

18. Cancer (keyword).

***or 19***

19. Neoplasms (MESH term).

***Limited to English language, human, all adult (19 plus years)***
